# Empagliflozin Ameliorates Progression From Prediabetes to Diabetes and Improves Hepatic Lipid Metabolism: A Systematic Review

**DOI:** 10.7759/cureus.28367

**Published:** 2022-08-25

**Authors:** Md Fahad Hossain, Nawsheen A Khan, Afroza Rahman, Mirza Farhana Iqbal Chowdhury, Sadia Bari, Mahfuza A Khan, Ummul Wara Masud, Ummul B Zakia, Shibani P Paul, Nishat Tasnim

**Affiliations:** 1 Hospital Medicine, Upazila Health Complex, Kishoreganj, BGD; 2 Pediatric, Caribbean Medical University, Chapel Hill, USA; 3 Hospital Medicine, Satkhira Medical College, Satkhira, BGD; 4 Hospital Medicine, Sylhet MAG (Muhammad Ataul Goni) Osmani Medical College Hospital, Sylhet, BGD; 5 Internal Medicine, Sher-E-Bangla Medical College, Barishal, BGD; 6 Internal Medicine, Sylhet MAG (Muhammad Ataul Goni) Osmani Medical College Hospital, Sylhet, BGD; 7 Internal Medicine, Sir Salimullah Medical College, Dhaka, BGD; 8 Internal Medicine, Comilla Medical College Hospital, Chittagong, BGD; 9 Hospital Medicine, Sacramento VA (Veterans Affairs) Medical Center, Mather, USA

**Keywords:** obesity, hepatic steatosis, insulin resistance, hepatic lipid metabolism, non-alcoholic steatohepatitis (nash), pre-diabetic and non-diabetic, non-alcoholic fatty liver disease (nafld), sglt 2 inhibitor, empa

## Abstract

Diabetes mellitus (DM) and hepatic steatosis are two of the most common metabolic syndromes that affect the health of people globally. Empagliflozin (EMPA) is a promising drug of choice for the diabetic population. Recent studies have shown its beneficial effects not only on diabetic patients but also on patients suffering from cardiac, hepatic, neurological, or pancreatic anomalies. In this paper, we systematically searched electronic databases to compile literature that focuses on EMPA’s effect on the prediabetic population, diabetic population, and hepatic lipid metabolism. We focus on the mechanism of EMPA, specifically by which it increases insulin sensitivity and fat browning and reduces fat accumulation. Overall, we hypothesized that by its effect on weight loss and reducing inflammatory markers and insulin resistance (IR), EMPA decreases the rate of prediabetes to diabetes conversion. We concluded that by improving hepatic and serum triglyceride, decreasing visceral fat, and its positive impact on hepatic steatosis, the drug improves hepatic lipid metabolism. Further research should be done on this matter.

## Introduction and background

The World Health Organization (WHO) claims that diabetes affects around 422 million people and accounts for 1.5 million annual deaths worldwide [1]. With such an increasing rate, manufacturing therapeutic drugs that primarily target hyperglycemia is in demand. Empagliflozin (EMPA), a sodium-glucose cotransporter 2 inhibitor (SGLT-2), is one such group of drug that blocks sodium-coupled glucose transporters, situated on the S3 segment's proximal convoluted tubule's apical membrane, and reabsorbs 90% of the blood's glucose [2,3]. Heise et al. conducted a placebo-controlled, randomized, double-blind trial to examine the pharmacokinetics and pharmacodynamics of EMPA [4]. Plasma glucose was lowered without any episodes of hypoglycemia, and no safety concerns were found in the amount of urine or micturition frequency and creatinine clearance. The study also demonstrated that the medication prevents the kidneys from reabsorbing 39%-64% of the glucose under constant settings [4].

The purpose of this paper is to demonstrate how EMPA ameliorates progression from prediabetes to diabetes and improves hepatic lipid metabolism. The mechanisms by which EMPA prevents diabetes are weight loss, changes in inflammatory markers, and reduction in insulin resistance (IR) and insulin amount. The conversion of white adipocyte tissue (WAT) to brown adipocyte tissue (BAT) via a mechanism called the browning process is a major target for obesity prevention. EMPA has a significant role in the process through its effect on mitochondrial biosynthesis in the AMP-activated protein kinase (AMPK) pathway, which leads to weight loss [5,6]. It upregulates the genes, *Ucp1*, *prdm16*, *Irisin*, *TFAM*, and *NRF2*, which are key players in the browning process [6]. By reducing inflammatory markers, EMPA reduces low-grade inflammation which contributes to IR [7-9]. Converting inflammatory macrophages to noninflammatory ones and decreasing the secretion of cytokines from macrophages and adipocytes are other mechanisms via which EMPA regulates inflammation to decrease IR. This paper also sheds light on studies that have demonstrated various mechanisms via which EMPA decreases IR [7] and restores hypothalamic insulin sensitivity in animal models, all of which contribute to reducing the rate of diabetes.

EMPA has a significant effect on hepatic lipid metabolism. This paper mainly focuses on how EMPA improves hepatic lipid metabolism by targeting hepatic triglyceride, serum triglyceride, and visceral fat and improves hepatic steatosis. We compiled literature that demonstrates how EMPA ameliorates diabetes and improves hepatic metabolism, by focusing on the pathophysiology of the disease in relation to the pharmacokinetics and pharmacodynamics of the drug. In the result section, a comparison table has been provided that demonstrates the limitations and outcomes of the various kinds of literature. Finally, we concluded our study by summarizing the findings and providing the strengths and limitations of our study.

## Review

Methodology

**Figure 1:**
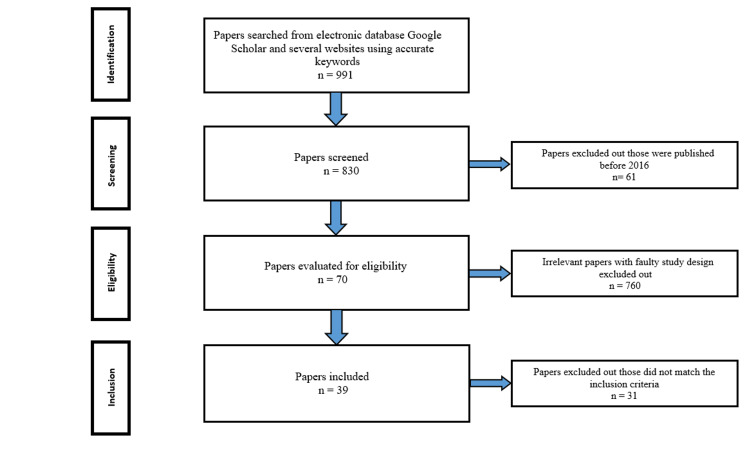
Flowchart showing PRISMA guidelines. Saha and Saha [10]. PRISMA: Preferred Reporting Items for Systematic Reviews and Meta-Analyses.

In this paper, we provided a systematic review of various kinds of literature on EMPA, using keywords based on our inclusion and exclusion criteria. Electronic database, Google Scholar, and a WHO website were searched with keywords such as EMPA, SGLT-2 inhibitor, prediabetic and nondiabetic, nonalcoholic fatty liver disease (NAFLD), nonalcoholic steatohepatitis (NASH), hepatic lipid metabolism, IR, hepatic steatosis, and obesity. A total of 991 (990 from Google Scholar and one from the WHO website) articles have been found. All these articles with their references have been screened and cross-examined cautiously to steer clear of any identical study. During eligibility assessment, papers that are complemented with the inclusion criteria have been added. Inclusion criteria were relevant to our study question, English language full-text paper published in the last seven years (2016-2022). A distinct variety of research papers have been embraced in our articles such as experimental studies on both humans and animals; randomized, double-blinded controlled trials; and review articles. However, papers written in different languages, published before 2016, books, case reports, and series have been excluded. After extensive filtering and analysis, studies with a faulty design that are not related to our research question and inclusion criteria have been eliminated. Finally, 39 articles have been selected and incorporated into our paper. The screening was done following the Preferred Reporting Items for Systematic Reviews and Meta-Analyses (PRISMA) protocol. It is shown in Figure 1 [10].

Relevant data from different studies were extracted and plotted into an Excel (Microsoft Corporation; Redmond, Washington, United States) spreadsheet. It consisted of study title, lead author and DOI, year of publication, study design, duration and population, interventions during experiments, study subjects and patient’s particulars, research outcomes, and the next research questions. Different study outcomes have been incorporated into our research such as visceral fat, serum triglyceride (s.TG), fat browning, hepatic steatosis, IR, weight loss, changes in the inflammatory markers, and all others.

Result

After reviewing the full text of the 39 papers, we have considered including six papers that contain answers to all our research questions for giving some important outcomes of EMPA treatment in a table. Here, we have added findings from one animal trial and five human studies (Table 1).

**Table 1:** Summary table of six important trials. EMPA: empagliflozin; BMI: body mass index; T2DM: type 2 diabetes; Nrf2: nuclear factor erythroid 2-related factor 2; NAFLD: nonalcoholic fatty liver disease; RCT: randomized controlled trial; NMR: nuclear magnetic resonance; VAT: visceral adipose tissue; FG: fasting glucose; qd: quater die sumendus (four times a day).

Study	Components	Features
Huttl et al., 2021 [11]	Study design	A randomized animal trial. The trial was conducted in the Czech Republic and accepted by the animal protection law of the Czech Republic.
	Summary	Eight prediabetic hereditary hypertriglyceridemia (HHT) and eight HHT rats were maintained at 12 hours dark/light circles at 22 degrees Celsius (C) temperature with adequate food and water. Rats were distributed into groups randomly with or without 10 mg/kg body weight EMPA for successive eight weeks. The experiment showed that EMPA decreased hepatic triacylglycerols and intermediates of lipotoxicity.
	Population	Total 16 rats. Control group: eight Wister male rats (six months old); Nonobese prediabetic model: eight HHT male rats (six months old).
	Intervention	Placebo and reference therapy (10 mg/kg body weight EMPA).
	Outcome	After administration of EMPA, beneficial improvements happened in insulin signaling and metabolism of lipid by alterations of cyt P450 proteins (Cyp 1a1, Cyp 2e1, Cyp 4a1, Cyp 4a2), genes (*Srebp 1*, *Pparγ*, *Scad 1*, *Fas*), and transcription factor (ex Nrf2).
	Limitation	The short period of time.
Kullman et al., 2022 [12]	Study design	A randomized, double-blind trial. The trial was supervised at the University Hospital of Tübingen.
	Summary	A total of 40 prediabetic patients participated with a BMI of more than 27, aged more than 50 years, and fasting glucose of greater than 5.41 mmol/L. The experimental group received 25 mg EMPA four times a day, whereas the control group received a placebo for successive eight weeks. There was a reduction in intrahepatic fat and full body adipose tissues in the EMPA group than in the control group.
	Population	Control group: 20 patients; Experimental group: 20 patients.
	Intervention	25 mg/kg body weight EMPA four times a day and placebo.
	Outcome	EMPA notably uplifted insulin responsiveness in the hypothalamus resulting in reduced hepatic fat as well as fasting blood sugar.
	Limitation	Small sample size. Different sexes were not equally distributed, so the sex-specific outcome was not available. Drug effects were measured only in the morning.
Hoda Taheri, 2020 [13]	Study design	A randomized, placebo-controlled, double-blind trial. Iran University of Medical Sciences approval.
	Summary	A total of 90 patients attended with NAFLD (without T2DM), variable ages from 20 to 65 years old, and BMI of more than 40 kg/m^2^. 10 mg/day of EMPA was taken by patients in the experimental group, and a placebo tablet was taken by the placebo group for 24 weeks. After the trial, there was a remarkable decline in BMI, waist circumference, weight, fasting insulin level, aspartate transaminase level, and alanine transaminase level in the EMPA group. 9.3% of this group fully recovered from fatty liver.
	Population	Control group: 47 patients; Experimental group: 43 patients.
	Intervention	10 mg/day EMPA and placebo tablet.
	Outcome	Liver stiffness measurement and liver fat were decreased notably in EMPA groups. Also, there was an improvement in hepatic steatosis status in patients with marked steatosis.
	Limitation	Liver biopsy is not the gold standard method for evaluating NAFLD status. Controlled attenuation parameter (CAP) cutoffs are lower, whereas transient elastography is being used nowadays for the detection of steatosis, as it has higher cutoffs.
Mantovani et al., 2020 [14]	Study design	Meta-analysis of RCTs.
	Summary	Twelve RCTs (six testing efficacy of dapagliflozin, three RCTs on EMPA, two RCTs testing ipragliflozin, and one RCT on canagliflozin). The drugs were to treat obese, middle-aged individuals with NAFLD. It was found that the drug decreased the amount of serum alanine aminotransferase, γ-glutamyl transferase, and the liver fat content percentage examined by magnetic resonance-based techniques.
	Population	850 individuals who had NAFLD, age 57 ± 6 years.
	Intervention	Placebo or reference therapy.
	Outcome	The drug showed reduced body weight and improved hemoglobin A1c levels.
	Limitation	In a small sample size of placebo-controlled and head-to-head RCT, 90% of the study population having both NAFLD and T2DM (only one patient had NAFLD without T2DM) RCTs with liver histological endpoints were included. MRI-based techniques are not an efficient method of determining treatment outcomes.
Lee et al., 2022 [15]	Study design	A randomized, placebo-controlled, double-blind trial.
	Summary	The goal was about studying the outcome of EMPA on blood triglycerides levels in the obese, nondiabetic population. Patients were given either placebo or EMPA for three months. After treatment and overnight fasting, they were given oral glucose. The goal of this study was to measure glycerol incorporation into triglycerides using NMR spectroscopy.
	Population	35 patients with a BMI of more than 30 kg/m^2^ were given either placebo or 10 mg/day EMPA for three months.
	Intervention	Participants were randomly given either 10 mg/day of EMPA orally or a placebo.
	Outcome	Triglycerides in the obese population with low visceral adipose tissue (VAT) were lowered, whereas triglycerides increased in obese subjects with high VAT. Triglyceride synthesis was reduced in low VAT obese subjects, not in those who had high VAT. Moreover, reduced body weight was also observed in obese subjects with high VAT.
	Limitation	Short period of treatment (only three months).
Hummel et al., 2020 [16]	Study design	Randomized, placebo-controlled, double-blind trial.
	Summary	Subjects were chosen randomly and given either placebo or EMPA for successive eight weeks. Before and after the trial, they participated in a 75 g oral glucose tolerance test in order to assess their glucose tolerance, peripheral insulin sensitivity, and insulin secretion. Total body fat, intrahepatic fat, and respiratory quotient (RQ) along with resting energy expenditure were estimated using whole-body MRI, indirect calorimetry, and localized MR spectroscopy, respectively.
	Population	40 subjects with prediabetic status. Age: 60 ± 9 years; BMI: 31.5 ± 3.8 kg/m^2^; FG: 5.98 ± 0.57 mmol/l.
	Intervention	25 mg EMPA qd or placebo were given randomly.
	Outcome	Total adipose tissue, RQ, and intrahepatic fat were lower in patients who were given EMPA which can be beneficial for obese prediabetic individuals with hepatic steatosis without affecting glucose level in the blood.
	Limitation	Small sample size.

**Figure 2:**
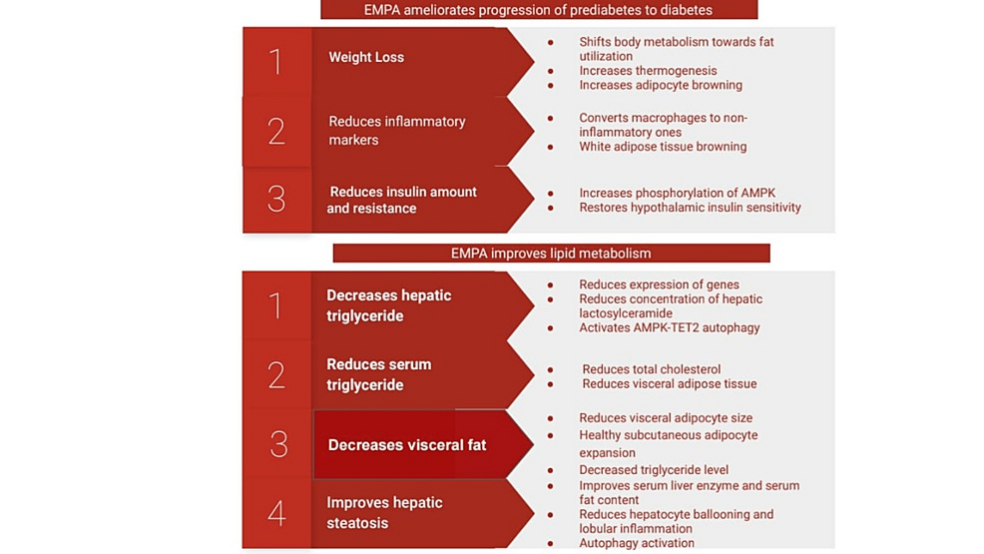
Summary of the mechanisms impacted by EMPA. Munir and Davis [3]. EMPA: empagliflozin; AMPK: AMP-activated protein kinase; TET2: tet methylcytosine dioxygenase 2.

All the studies included here are placebo-controlled. The duration of studies ranged from eight weeks to three months, including three that have the same eight weeks endpoints. Overall, the mentioned human studies were comparable in aspects of age, body mass index (BMI), and hepatic lipid status as the criteria of the patient for inclusion in human studies, whereas in animal studies, prediabetic hepatic lipid status was the selection criterion. In general, a low risk of bias is observed. In most of the studies, it is notable that EMPA improved hepatic lipid metabolism, hepatic steatosis, insulin sensitivity, fasting glucose level, and reduced body weight in some studies. Although there are reasonable amounts of animal trials available, slender amounts of human trials and shorter periods of study duration can be proved as major drawbacks. In Figure 2, we summarize the mechanism of EMPA [3].

Discussion

As the commonest form of diabetes mellitus, type 2 diabetes (T2DM) is becoming more and more prevalent each day. Many factors like obesity, an increase in the inflammatory markers in the insulin target tissue, and an increase in IR and amount are associated with developing T2DM. Obesity is a rising concern for NAFLD also. EMPA, a relatively new antidiabetic drug, has been proven as an excellent choice in controlling diabetes mellitus (DM) [17]. It also augments the effect of insulin [18]. Many similar studies have been done to find out the differences in outcome in different pharmacokinetics and pharmacodynamics of EMPA [4,19]. Also, different research has been carried out to find out the action of the SGLT-2 inhibitor beyond its standard measure of controlling blood glucose in DM [3,20-25]. However, recent studies have revealed that EMPA acts on the various factors that lead to the development of diabetes. EMPA was also found to improve the beta cell function in impaired fasting glucose patients [26]. Thus, EMPA has the potential to delay the development of DM. It also has several actions on lipid metabolism. In our systematic review, we have tried to show how EMPA improves hepatic lipid metabolism and ameliorates the progression of prediabetes to diabetes.

Diabetes Prevention

EMPA affects the following mechanisms in its role in diabetes prevention: a) weight loss; b) change in the inflammatory markers; and c) reduction of IR and amount.

Weight loss: The relationship between obesity and T2DM has been recognized for several years. The IR of various target tissues, such as skeletal muscle, adipose tissue, and the liver, is exacerbated by obesity. Additionally, the majority of T2DM patients are obese. Reduced prevalence of diabetes and metabolic syndrome is linked to weight loss. In their experimental study, Xu et al. [27,28] found that high feed diet (HFD) male C57BL/6Jslc mice that are given 0.01% (w/w) EMPA for eight weeks and 16 weeks manifested reduced weight gain compared to control. Human trials support this result [16,29], which showed a relationship between loss in body adipose tissue and hepatic fat content [16]. A possible mechanism of body weight loss is shifting the body's energy metabolism toward utilizing fat content. Also, EMPA increases thermogenesis as evidenced by the increase in the level of protein uncoupling protein 1 (UCP-1) in both brown and white adipose tissues [27]. This mechanism is helpful as, theoretically, it would utilize more energy without storing it. EMPA also increases adipocyte browning [5], which has more thermogenesis potential, as brown adipose tissue has mitochondria that produce heat in the respiratory chain instead of making energy. All those mechanisms assist in lowering body weight.

Change in the inflammatory markers: Low-grade inflammation, which is related to elevated production of inflammatory cytokines like tumor necrosis factor (TNF), interleukin 6 (IL-6), and leptin, is one of the typical pathways of the onset of IR in DM patients. Several preclinical studies have shown that EMPA reduces low-grade inflammation by reducing inflammatory markers [7-9,30]. Hupa-Breier et al. [31] found a decrease in inflammation with the combined use of dulaglutide and EMPA, although no beneficial effect of EMPA alone was seen. Several mechanisms are thought to be the cause of this change. One mechanism is that, by directly acting on the macrophage and adipose tissue, EMPA decreases the secretion of inflammation markers. EMPA also stimulates the conversion of macrophages to noninflammatory ones. White adipose tissue (found in higher amounts in obese patients) contains more macrophages than brown adipose tissue. EMPA plays a significant role in white adipose tissue browning, which not only decreases obesity but also lowers the rate of chronic inflammation.

Reduction of IR and amount: The development of IR is the initial process that ultimately leads to DM. Different mechanisms are responsible for IR. Various experimental research works have been carried out to demonstrate the efficacy of different drugs in reducing IR and preventing diabetes. EMPA showed promising results in this field. An experimental study with high-fat diet (HF) male C57Bl/6 mice that are given 10 mg/kg/day of EMPA for five weeks showed significant IR and amount [7]. In addition to indirectly improving IR, EMPA seems to increase the phosphorylation of the AMPK enzyme in HF mice which is an important sensitizer of the cellular energy balance. Moreover, a study involving human subjects by Kullmann et al. [12] found that treatment with EMPA for eight weeks restored hypothalamic insulin sensitivity in the prediabetic patient. Furthermore, another study of SGLT-2 inhibitors on overweight, nondiabetic insulin-resistant persons supported the previous study by showing reduced IR and amount following 12 weeks of experiment [23,32].

Effects on Lipid Metabolism

NAFLD is a well-established association of insulin-resistant conditions, including T2DM. In this systematic review, we tried to determine the factors responsible for NAFLD development and how EMPA modifies them. NAFLD pathophysiology depends on multiple constituents like hepatic lipid accumulation, IR, oxidative stress, endoplasmic reticulum stress, and other injuries. EMPA is found to be efficacious in minimizing all these aspects and thus reducing the NAFLD development [33-35].

Hepatic triglyceride: NAFLD occurs due to the collection of triglyceride (TG) in the cytosol of liver cells and also due to a disparity between lipid collection and lipid elimination. Lipid acquisition occurs by uptake of fatty acids (FAs) and utilizes them for the synthesis of TG, whereas elimination occurs by oxidation in mitochondria which are transported as a constituent of very low-density lipoprotein (VLDL) particles. Researchers found that EMPA administration causes a decrease in ectopic triacylglycerol and lipotoxic diacylglycerol accumulation in the liver [36]. It has also reduced the mRNA expression of fatty acid synthetase (fas) and stearoyl-CoA desaturase 1 (scd1), which are lipogenic enzymes. EMPA treatment reduces the expression of sterol regulatory element-binding protein 1 (srebp1) and peroxisome proliferator-activated receptor-α (PPAR-α), and thus, it decreases the hepatic lipid accumulation by suppressing hepatic lipogenesis [11]. Scd1 is found to be decreased in other studies as well [7,37]. In this way, it improves hepatic insulin sensitivity and thus reduces the progression to NAFLD. Xu et al. have found that EMPA reduces the concentration of intrahepatic lactosylceramide and increases the unsaturated triglycerides; thus, it helps in exerting beneficial effects in metabolic and hepatic pathways in nondiabetic mice [27].

Serum triglyceride: IR and T2DM are related to high serum triglyceride levels. EMPA is found to be effective in reducing serum triglyceride levels. Nasiri-Ansari et al. found that EMPA reduces the level of total cholesterol, as well as serum triglyceride levels in high-fat diet-fed mice [37], while some researchers found that EMPA treatment reduces total cholesterol levels [7]. However, it has no effect on plasma triglyceride level [7]. In a randomized controlled trial, researchers have observed that EMPA response depends on the amount of visceral adipose tissue. EMPA treatment for three months reduced serum triglyceride only with low visceral adipose tissue. On the contrary, it increases serum triglyceride in patients with high visceral adipose tissue [15].

Hepatic steatosis: One of the first processes incorporated in the mechanism of hepatic steatosis (HS) is hepatic lipid accumulation and the eventual occurrence of IR. The next process is the inflammation and hepatic damage mediated by oxidative stress [11]. Recent studies suggest that EMPA treatment in T2DM patients significantly reduced liver fat content [22]. A meta-analysis of placebo-controlled RCT involving adult individuals with NAFLD suggests that SGLT-2 inhibitors cause a remarkable improvement in hepatic enzymes and also diminish liver fat content [5]. Nasiri-Ansari et al. [37] discovered that, after five weeks of treatment with EMPA, apolipoprotein E (ApoE) (-/-) mice had reduced expression of six key enzymes involved in the fatty acid synthesis. They also found that there was a remarkable reduction of HS, hepatocyte ballooning and degeneration, and lobular inflammation in the mice treated with EMPA. In a prospective, double-blinded, placebo-controlled RCT of EMPA in NAFLD patients without T2DM, 24 weeks of EMPA treatment notably decreased serum alanine aminotransferase (ALT), aspartate aminotransferase (AST), and liver fat content as compared to baseline [14]. A protective mechanism against HS is autophagy. Autophagosomes engulf intracellular lipid droplets, which subsequently undergo lysosomal degradation. In an experimental study involving db/db mice, EMPA increased the phosphorylation of AMPK, which is a major regulator of autophagy. This resulted in autophagy activation and decreased hepatic lipid deposition in vivo and in vitro. Research studies also established the AMPK-tet methylcytosine dioxygenase 2 (TET2)-autophagy signaling pathway, where the TET2 protein acts as an intermediary controller of the AMPK-autophagy pathway. Autophagy activity was diminished when the TET2 level was decreased in the cell. Study results also demonstrate that EMPA attenuates HS by activating AMPK, thereby maintaining intracellular TET2 protein levels and inducing autophagy [5].

Visceral fat: Body fat content has a critical impact on whole-body metabolism. Visceral and subcutaneous/peripheral fat are different types of fat according to the location within the body. Visceral fat can be associated with IR and dyslipidemia, whereas subcutaneous white fat may have some protective role against harmful lipid build-up in the body [23,38]. In their experimental study, Kurtz et al. [39] observed that EMPA-treated TallyHo mice had reduced visceral adipocyte size and decreased triglyceride level, along with healthy subcutaneous adipose expansion. This finding was supported by another experimental study, where SGLT-2 inhibitors were found to reduce body adipose tissue and hepatic fat content, possibly by making lipid oxidation as the major source of energy in the body [31].

In short, EMPA restores hypothalamic insulin sensitivity, improves hemoglobin A1c level in the prediabetic patients, and halts the progression of prediabetes to diabetes [12,14,32]. Several studies on humans and animals showed that EMPA reduces total cholesterol, s. TG, total body (especially intrahepatic) fat content, and accumulation. Thus, it prevents hepatocyte ballooning, degeneration, inflammation, and HS progression [5,16,22,31,37,39].

Strengths

This study was conducted according to the PRISMA guideline and reviewed a large number of articles before selecting 39. However, we did not restrict our research only to the studies done on human subjects, we added several animal studies too. We discussed two important metabolic diseases (DM and HS) and their treatment with a single medication, that is, EMPA. Furthermore, we explained the detailed mechanism of EMPA, by which it prevents progression to DM and HS.

Limitations

We specifically limited our study to obese patients. Therefore, we did not convey enough data on patients with normal weight. Also, we searched for articles in the Google Scholar database only, there might be some other articles related to our research hypothesis with different findings in other electronic databases. There were very few articles addressing the EMPA effect on prediabetes. Moreover, we did not explore the drug interactions and harmful effects caused by EMPA.

## Conclusions

EMPA, a comparatively newer drug, has manifested significant beneficial roles in treating both prediabetic and diabetic patients. In this article, we have discussed the mechanisms by which EMPA exerts different favorable effects, restores insulin sensitivity, and decreases total cholesterol, s. TG, total body fat content, fat accumulation, hepatic steatosis, and body weight. Therefore, it can be said that EMPA prevents the progression of prediabetes to diabetes. It has also been demonstrated to reduce the development of hepatic steatosis by increasing hepatic lipid metabolism. So, further research studies should be done on human subjects to identify other useful and harmful effects of EMPA.
